# Functional food for functional disorders

**DOI:** 10.1080/16512235.2017.1281955

**Published:** 2017-02-06

**Authors:** Arnold Berstad, Jan Raa, Jørgen Valeur

**Affiliations:** ^a^ Unger-Vetlesen Institute, Lovisenberg Diaconal Hospital, Oslo, Norway

Unexplained hypersensitivity to food is a major health problem affecting about 10% of the population, and is often associated with disabling symptoms, such as irritable bowel syndrome (IBS), musculoskeletal pain (fibromyalgia) and fatigue (chronic fatigue syndrome/myalgic encephalomyelitis; CFS/ME). Recent metabolomic studies suggest a hypometabolic state with excessive oxidative stress, impaired oxidative phosphorylation, and an energy production dependent on the metabolism of fat and amino acids.[1] ‘Missing microbes’ [2] following the use of antibiotics at an early age may be an important cause, possibly assisted by a Western diet high in simple carbohydrates.[3]

The present preliminary report is the result of a systematic survey of 438 consecutive patients examined by one of us (AB). Validated diagnostic criteria and scoring questionnaires for symptom severity were applied.[4] Briefly, the results are as follows. Age of the patients is on average 37 years; 2/3 are women. Virtually all patients have irritable bowel syndrome (IBS) with symptoms related to incomplete evacuation and altered intestinal fermentation. A selection of the patients who have undergone ultrasonography or MRI scans are found to have increased amount of fluid persisting in bowel loops one hour after the ingestion of lactulose, consistent with intestinal hypomotility.[5,6] Although perceived food intolerance was the ‘entrance ticket’ for medical investigation, IBS was rarely attributable to food allergy. In addition to IBS, about 70% of the patients had musculoskeletal pain and 85% chronic fatigue. About half of those with fatigue met the international criteria for an ME diagnosis, and this diagnosis correlated well with being placed on long-term sick leave. The history of illness averaged 26 years. The first symptoms were almost always related to abdominal discomfort, and very often (in about 90% of the cases) the symptoms appeared after prolonged antibiotic treatment at an early stage of life. Subsequently, the problems deteriorated, often after bouts of gastroenteritis and/or further courses of antibiotic treatment. Our results therefore suggest that both the perceived food hypersensitivity and IBS, acquired following antibiotics at early life, and the subsequently acquired musculoskeletal pain (including temporomandibular dysfunction), chronic fatigue and ME are aspects of *one* disease, not several different diseases.

Seminal studies show loss of lactic acid bacteria and increased growth of yeast (*Candida albicans*) in the bowel immediately following the use of antibiotics.[7,8] Previously, it was believed that this altered bowel flora would normalise in the course of a week. However, recent studies show that this is not always the case; sometimes the bowel flora never fully recover.[2,9] ‘Missing microbes’ following the use of antibiotics may lead to the growth of unfavourable flora, frequently in the form of a biofilm with yeast and facultatively anaerobic microbes.[10] This biofilm elicits little local damage, but stimulates innate immunity and the release of interferon gamma, which in turn activates indoleamine dioxygenase (IDO) and the metabolism of tryptophan along the kynurenine pathway.[11] The result is an altered immune response with increased immune tolerance, protecting the pathological flora.[12] However, tryptophan is also the precursor of important hormones such as serotonin and melatonin – and increased metabolism of tryptophan to kynurenines may lead to serotonin and melatonin deficiency.[13,14] Shortage of serotonin may explain poor intestinal peristalsis, sadness and ‘brain fog’, while melatonin deficiency may explain poor sleep and the feeling of never being completely refreshed. Increased physical and cognitive fatigue may largely be due to oxidative stress, possibly because a deficiency of lactic acid bacteria may result in excess oxygen levels within the bowel. By producing hydrogen peroxide, these microbes pick up remaining oxygen and thus help to maintain low oxygen tension. If the redox potential is too high, the metabolism of the host switches to an oxidative shielding response [15] with aerobic glycolysis and deficient mitochondrial energy (ATP) production (Crabtree effect).[16]

Some important aspects of the treatment are: dietary carbohydrate restriction may reduce IBS symptoms,[17] while commercial probiotic lactobacilli may do more harm than good.[18] Various forms of fecal microbiota transplantations are currently being investigated.[19,20] Because tryptophan appears to play such important microbial and immunological roles in functional disorders,[21] we find it an interesting option to provide tryptophan as tryptophan-rich peptides.[22,23] Simultaneously, we are very concerned about the wide use of the herbicide glyphosate (Roundup), which blocks the synthesis of tryptophan in plants and microbes [24] and thus impoverishes our natural resources of the essential tryptophan.
